# Rectal surgery in octogenarian patients: our experience

**DOI:** 10.1186/1471-2482-13-S1-A30

**Published:** 2013-09-16

**Authors:** Manuela Monni, Alfonso Terrone, Alberto Oldani, Clemente De Rosa, Marcello Garavoglia

**Affiliations:** 1 Department of General Surgery, “Maggiore della Carità” Hospital / University of East-Piedmont “Amedeo Avogadro”, Novara, Italy

## Introduction

Colo – rectal cancer is one of the most common tumors, especially in elderly patients; surgical procedures with curative or palliative intention in colo-rectal neoplasm in elderly patients due to the issue they raise: benefits versus increased mortality [1].

The aim of our study is to analyze patient’s outcome after surgery for rectal cancer and compare morbidity rate with a population of younger patients treated for the same pathology.

## Methods

We performed in our Department 213 consecutive surgical treatments for rectal cancer from March 1999 to May 2013: depending on the age, all patients were included in two different groups: Group I- patients younger than 75 years and Group II those of 75 years or older at median age of 68,7 years (range 43-93 years).

Rectal cancers treatable by elective surgery, underwent pre-operative staging firstly with colonoscopy and biopsy to establish the distance from the anal verge end and the histological pattern; then pre-operative staging was completed with chest X-ray, abdominal ultrasound and chest-abdominal CT or NMR, (gold standard to stage low rectal disease) to detect local and distant involvement and the presence of metastatic localizations.

Rectal cancers that needed urgent surgery generally were study only by TC-scan, in order to perform surgery as soon as possible, so no endoscopy was disposed.

Our experience reported that in both groups the more frequent tumor’s rectal location was upper rectum: in younger group was: 58 upper rectum (40.8%), 42 medium rectum (29.5%) and 42 low rectum (29.5%). In the elderly patients: 33 upper (46.4%), 17 medium (23.9%) and 21 low rectum (29.5%).

Patients anesthesiologic status and surgical risk were classified according to the American Society of Anesthesiologists classification of physical status (ASA class I-V), in our experience older people had an prevalence of ASA III, compared to more frequent ASA II of younger group.

Both two groups evidenced that T3N1M0 stage of disease is the more common (Group I 48.5% – Group II 46.4%), so patients could be candidate to curative surgery.

## Results

On the basis of preoperative staging, through clinical multidisciplinary discussion, 59 patients received neo-adjuvant therapy (radiotherapy alone or radiotherapy with chemotherapy).

Surgical therapy included radical surgery by performing Miles Abdominal Perineal Amputation (40 pts, Group I 24 and Group II 16), laparothomic (122 pts, Group I 92 and Group II 30), and laparoscopic (7 pts, Group I 7 and Group II 0) low anterior resection and Hartmann’s resection (21 pts, Group I 9 and Group II 12). Other radical surgical treatments were performed in 11 patients. Even if we performed different surgical options and different kind of anastomosis, mechanical anastomosis was the most performed (99%).

Palliative surgery (such as ileo-colostomy) were served for patients with no-eradicable disease, with peritoneal carcinosis or for very low performance status patients not able to effort an aggessive surgical treatment: we treated this way 12 patients (Group I 5 and Group II 7).

Focusing on post-operative management, we reported complete absence of complication for 121 pts (56.8%), with slight rate complications (as urinary retention, anemia, constipation) at 55.4% and 6.9% of serious surgical complication included anastomotic leaks, emorrhagic events, ureteral injury, bowel obstruction and necrosis of colostomy, all them required relaparotomy.

Comparing two groups, we notice that the overall percentage of complication was not so dissimilar in the two groups (47.8% in elderly people – 52.2% in younger patients) but it so important to evidence that complications very often occurred in people with past history of clinical comorbidities such as diabetes, cardiac failure, lung disease, so not related to the age.

## Conclusions

Literature doesn’t report a clear definition of “elderly patients”, so different authors in different studies, consider elderly people of 65, 70, 75, 80 years [2-5], by in our experience we selected the cut off of 75 years to divide our patients into two groups.

Lots of studies have demonstrated strong correlation between age, ASA scoring and post-operative outcome [5] and even in our experience we observed similar correlations, but we reported that 96% of our elderly patients underwent curative surgery.

The incidence of post-operative morbidity and complications are more frequent progressively with advancing age [3-5] but elderly patients with no serious comorbidities had regular post-operative management with no complication as younger people at the same clinical stage, otherwise it is interesting to notice that younger people with metabolic and chronic disease had worse clinical outcome compared to healthy aged patients.

So our conclusion is that stage of disease at diagnosis remains the major determinant of prognosis and that advanced age alone is not a contraindication to radical surgery and that elderly group can also benefits from adjuvant therapies with good overall and disease free survival.
